# *NFKB1* gene rs28362491 ins/del variation is associated with higher susceptibility to myocardial infarction in a Chinese Han population

**DOI:** 10.1038/s41598-020-72877-9

**Published:** 2020-11-11

**Authors:** Jun-Yi Luo, Yan-Hong Li, Bin-Bin Fang, Ting Tian, Fen Liu, Xiao-Mei Li, Xiao-Ming Gao, Yi-Ning Yang

**Affiliations:** 1 State Key Laboratory of Pathogenesis, Prevention and Treatment of High Incidence Diseases in Central Asian, Department of Cardiology, First Affiliated Hospital of Xinjiang Medical University, 137 Liyushan South Road, Urumqi, 830054 Xinjiang China; 2 Department of Medical Science Examination Center, First Affiliated Hospital of Xinjiang Medical University, Urumqi, Xinjiang China; 3 State Key Laboratory of Pathogenesis, Prevention and Treatment of High Incidence Diseases in Central Asian, First Affiliated Hospital of Xinjiang Medical University, 137 Liyushan South Road, Urumqi, 830054 Xinjiang China; 4 Xinjiang Key Laboratory of Medical Animal Model Research, Urumqi, China

**Keywords:** Myocardial infarction, Genetic linkage study

## Abstract

Myocardial infarction (MI), the leading cause of mortality and disability worldwide, is a disease in which multiple environmental and genetic factors are involved. Recently, researches suggested that insertion/deletion (ins/del) variation of *NFKB1* gene rs28362491 is a functional polymorphism. In the present study, we aimed to explore the relation between variation of *NFKB1* gene rs28362491 and MI by polymerase chain reaction–restriction fragment length polymorphism (PCR–RFLP) in 359 MI patients and 1085 control participants. Gensini score was used to evaluate the degree of coronary artery stenosis in MI patients. The plasma levels of interleukin-6 (IL-6), IL-8, malonaldehyde (MDA) and superoxide dismutase (SOD) were randomly measured by ELISA both in MI patients and control participants. We found that the detected frequencies of D allele (41.2% vs. 36.4%, *P* = 0.021) and DD genotype (17.5% vs. 12.0%, *P* = 0.022) were significantly higher in MI patients than in control participants. Compared with II or ID genotype carriers, the Gensini score in MI patients with DD genotype was 32–43% higher (both *P* < 0.001). Moreover, DD genotype carries had more diseased coronary arteries (*P* = 0.001 vs. II or ID genotype). Of note, IL-6 levels in MI patients carrying DD genotype were significantly higher than that in control participants and other genotype carriers in MI patients (both *P* < 0.05). In conclusion, *NFKB1* gene rs28362491 DD genotype was associated with a higher risk of MI and more severe coronary artery lesion, which also had a potential influence on the level of inflammatory cytokine IL-6.

## Introduction

Myocardial infarction (MI), a leading cause of mortality and disability worldwide, is due to necrosis of ischemic myocardium which caused by occlusive thrombosis in the coronary artery^1,2^. Except well-known traditional risk factors such as dyslipidemia, high blood pressure, diabetes, obesity, smoking, physical inactivity and age, recently, genetic factors including angiotensin-converting enzyme (ACE) gene^3^, serotonin transporter (SLC6A4) gene^4^, endothelial nitric oxide synthase (eNOS) gene^5^ have been reported to link with the risk of MI. In addition, different genetic variation of a single gene such as base substitution, translocation, deletion and insertion has also been found to associate with the susceptibility of MI^6^.

The nuclear factor kappa B family (NF-κB) is a vital and widespread transcription factor and usually exists in the cytoplasm in eukaryotic cells^7^. NF-κB transfers into the nucleus upon activation and bind to promoter regions of proinflammtory genes such as interleukin-6 (IL-6) IL-8 and TNF-α^8,9^, which influences broad biological processes such as energy metabolism, cell apoptosis and proliferation, inflammation. It is also one of the cellular sensors which responds to oxidative stress and regulates gene expression including malonaldehyde (MDA) and superoxide dismutase (SOD), these are measurable markers of oxidative stress^10–12^. In mammals, there are five key subunits in NF-κB family containing p50/p105, p65, c-Rel, RelB and p52/p100. All these subunits share a highly conserved DNA-binding/dimerization domain called the Rel homology domain^13^. The p50 and p52 transcription factors, derived from precursor proteins p105 and p100, respectively, lack transcriptional activation domains, and their homodimers are thought to act as transcriptional repressors^14^.

*NFKB1* gene rs28362491 encodes p50/p105 submits of NF-κB family and locates at chromosome 4q24. A four base ATTG insertion/deletion (ins/del) variation located in −94 loci of *NFKB1* promoter has been reported, which encodes three genotypes: wild-type homozygous insertion (II genotype), variant homozygous deletion (DD genotype) and heterozygous (ID genotype)^15^. Studies have shown that individuals who carried *NFKB1* gene rs28362491 D allele or DD genotype were more susceptible to inflammatory diseases such as inflammatory bowel disease^16^, ulcerative colitis^17,18^ and atherosclerotic cardiovascular diseases including coronary artery disease (CAD)^19–21^ in different populations. However, a small clinical study (86 MI patients and 167 controls) observed opposite results. It showed that the frequencies of D allele and DD genotype of *NFKB1* gene rs28362491 were much lower in MI patients than that in controls. It also observed that individuals carrying D allele had lower plasma levels of fibrinogen and C-reactive protein and lower myocardial p50 level compared to no D allele carriers^22^. Others found the frequencies of D allele and DD genotype were comparable between MI patients and controls^23^.

It has been clearly documented that myocardial ischemia can trigger nuclear translocation of NF-κB which in turn regulates expression of a variety of downstream genes^24^. Considering inconsistent results and the well-known physiological relevance of NF-κB in atherosclerotic and inflammatory diseases, we decided to conduct a case–control study to investigate the relation between *NFKB1* gene rs28362491 variation and MI susceptibility in a relatively large population. We also assessed the severity of coronary artery lesions. Circulating levels of inflammatory cytokine and oxidative stress biomarkers were also measured to explore the functional influence of this variation.

## Materials and methods

This study was approved by the Ethics Committee of the First Affiliated Hospital of Xinjiang Medical University according to the standards of the Declaration of Helsinki. Written informed consents were obtained from all participants.

### Study population

This was a case–control study to investigate the relation between *NFKB1* gene rs28362491 variation and MI susceptibility in Chinese Han population in Xinjiang, northwest of China. All the participants were recruited at the First Affiliated Hospital of Xinjiang Medical University from 2010 to 2014. Diagnosis of MI was made according to the guidelines such as a plasma creatine kinase-MB level > 2-fold than the normal value or a cardiac high-sensitivity troponin T (hs-TnT) > 0.1 mg/mL after symptom onset together with at least 1 of the following: (1) chest pain persisting for > 20 min; (2) electrocardiograph exhibiting elevation of the ST segment > 1 mm or a new pathological Q wave^25^. All MI participants underwent arteriography to verify coronary artery stenosis (> 50% reduction luminal diameter). We also recruited control participants who had no history of cardiovascular diseases, and no signs of ischemic heart disease based on the guidelines. The exclusion criteria for all participants were those with valvular heart disease, congenital heart disease, non-ischemic cardiomyopathy, or acute and chronic inflammatory diseases.

### Analysis of coronary stenosis degree

Gensini score was calculated according to the angiography result to evaluate the extent of coronary stenosis^21,26^. Briefly, the stenotic coronary arteries in the range of 1–25%, 26–50%, 51–75%, 76–90%, 91–99%, as well as complete occlusion (100%) were scored as 1, 2, 4, 8, 16, and 32 points, respectively. Furthermore, a multiplying factor representing the functional significance of the area supplied by each stenotic coronary artery. Specifically, the factor of 5 for left main coronary artery; 2.5 for the proximal segment of left anterior descending (LAD) and circumflex artery (LCX); 1.5 for the middle segment of LAD; 1 for the first diagonal branch, the distal segment of LAD, the obtuse marginal branch, the proximal, middle or distal segment of the right coronary artery (RCA), the posterior descending artery, the distal segment of LCX, and left ventricular posterior branch; and 0.5 for other arteries. The total Gensini score for each MI patient was the sum of each individual score corresponding to related stenotic artery. We also calculated the number of diseased arteries (> 50% narrowing of the lumen diameter).

### Biochemical analysis

Peripheral venous blood samples (5 mL) were collected in EDTA-containing tubes from all participants following overnight fasting for biochemical assays. Subsequently, the samples were centrifuged at 5000 rpm for 5 min to separate plasma from blood cells at 4 °C. Part of plasma samples were sent to the Central Laboratory of First Affiliated Hospital of Xinjiang Medical University for biochemical assays including glucose, total cholesterol (TC), triglycerides (TG), low density lipoprotein-cholesterol (LDL-C) and high density lipoprotein-cholesterol (HDL-C) using the commercially available automated platform. Blood cells were kept at −80 °C separately for further analysis.

### Definition of cardiovascular risk factors

Body mass index (BMI) was calculated by dividing body weight (in kilograms) with the height in meters squared. Individuals who smoked regularly in the past 6 months were regarded as current tobacco users. Hypertension was defined as systolic blood pressure (SBP) exceeded 140 mmHg and/or diastolic blood pressure (DBP) was more than 90 mmHg on at least two different occasions^27^. A person who had abnormal fasting blood glucose or an abnormal glucose tolerance was defined as the diabetes patient according to the World Health Organization criteria^28^.

### Genetic polymorphism detection

Genomic DNA was extracted from the peripheral leukocytes using a whole blood genome extraction kit (Boiteke Corporation, China). The *NFKB1* gene rs28362491 −94 ATTG ins/del variation was detected by polymerase chain reaction–restriction fragment length polymorphism (PCR–RFLP) according to our previous study^26^. The primers for PCR amplification were as follow: 5′-TGGGCACAAGTCGTTTATGA-3′ (forward) and 5′-CTGGAGCCGGTAGGGAAG-3′ (reverse). PCR product was digested with 1 unit of restriction enzyme *PfIMI* (Fermentas, Canada). The PCR product-enzyme mix was incubated overnight at 37 °C and subsequently ran on electrophoresis for 30 min at 100 V in 2% agarose gel.

### Enzyme linked immunosorbent assay (ELISA)

To investigated whether *NFKB1* gene rs28362491 variation had an influence on NF-κB related factors, we randomly measured plasma levels of IL-6, IL-8, MDA and SOD by commercial ELISA kits (Cloud-Clone Corp, USA) according to the manufacturer’s instructions in 75 MI patients and 124 control participants. The randomization of the participant selection for ELISA was computer generated at the Key Laboratory of Cardiovascular Disease Research of Xinjiang Medical University (blocked randomization) and the participants' allocations were kept in sequentially numbered and sealed envelopes. The steps were as follows: first, all the controls and MI patients were numbered as 1–1085 and 1–359, respectively. Second, we divided controls and MI patients separately into blocks containing five participants. Third, we use random number table to identify the number for the first individual selected from any block both in controls and MI patients, respectively. Last, for the second sample, its number was the number of the first patient plus five from the following block. Sampling was carried out following the rule in turn until the required sample size was obtained.

### Statistical analysis

Data analysis was performed using Statistical Package for Social Sciences-SPSS (version 17.0, SPSS Institute, Chicago, IL, USA). Hardy–Weinberg equilibrium was assessed by Chi-square test. Continuous variables were expressed as mean ± standard deviation (SD) and the difference between MI and control groups was detected by independent-sample *t* test. Two-way ANOVA were used to analysis the difference of levels of IL-6, IL-8, MDA and SOD in participants with different genotypes. Categorical variables were presented as numbers or percentages and the difference between two groups was detected by Chi-square test. The univariate logistic regression analysis was used to evaluate the association between each variables and MI. All variables in the univariate analysis which yielded *P* < 0.05 were selected as candidate for a multivariate logistic regression analysis to determine any independent predictors of MI. Crude odds ratios (COR) and adjusted odds ratios (AOR) were calculated along with 95% confidence interval (CI). The power of this study was calculated by Power and Sample Size Program (Version 3.0.43)^29^. *P* value < 0.05 was deemed statistically significant.

### Consent for publication

All authors have read the paper and approved this submission.

## Results

### Characteristics of the study population

We compared some traditional risk factors such as age, sex, smoking, levels of glucose and lipid in plasma between MI patients and controls (Table 1). We found that MI patients had higher levels of glucose, TC and LDL-C and lower HDL-C level compared with control group (all *P* < 0.05). The prevalence of male, smoking, hypertension and diabetes was also higher in MI patients than in control group, while age, BMI and TG level were comparable between the two groups (all *P* > 0.05).

**Table 1:** Clinical characteristics of study participants.

	Controls (n = 1085)	MI patients (n = 359)	*P* value
Age (years)	58.5 ± 11.3	59.3 ± 10.6	0.28
Male (n, %)	610 (56.2)	249 (69.4)	< 0.001
BMI (kg/m^2^)	25.7 ± 3.2	25.8 ± 2.9	0.726
Smoking n (%)	317 (29.2)	168 (46.8)	< 0.001
Glucose (mmol/L)	5.2 ± 1.5	5.7 ± 1.6	< 0.001
TC (mmol/L)	4.4 ± 0.6	4.6 ± 0.7	< 0.001
TG (mmol/L)	1.5 ± 0.6	1.5 ± 0.5	0.92
LDL-C (mmol/L)	2.4 ± 0.5	2.5 ± 0.6	< 0.001
HDL-C (mmol/L)	1.1 ± 0.2	1.0 ± 0.2	0.019
Hypertension n (%)	133 (12.3)	65 (18.1)	0.005
Diabetes n (%)	31 (2.9)	46 (12.8)	< 0.001

Continuous data were presented as mean ± SD. Categorical data were presented as the number (percentage).

*MI* myocardial infarction, *BMI* body mass index, *TC* total cholesterol, *TG* triglycerides, *LDL-C* low density lipoprotein-cholesterol, *HDL-C* high density lipoprotein-cholesterol.

### Distribution of *NFKB1* −94 ATTG polymorphism

**Figure 1:**
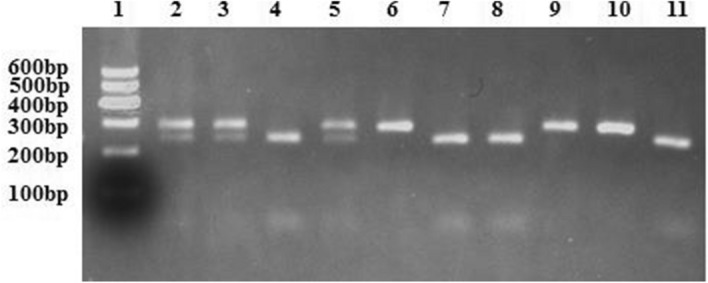
Polymerase chain reaction-restriction fragment length polymorphism analysis for genotyping of *NFKB1* rs28362491 variation. Lane 1 is a 600 bp DNA marker; lanes 2, 3 and 5 are ID genotype, lanes 4, 7, 8, and 11 are II genotype; and lane 6, 9, and 10 are DD genotype.

As the genomic DNA with D allele do not contain *PflMI* restriction site, the PCR products from participants carrying DD genotype could not be digested by *PflMI* incision enzyme, which appeared on the location of 281 bp in the electrophoretic gel imaging. For the PCR products from II genotype, they were cleaved into two fragments of 240 and 45 bp by *PflMI*. The PCR products from heterozygote ID genotype were digested into three bands of 285 bp, 240 bp, and 45 bp (Fig. 1, the original result was showed in Supplementary Figure S1). Distribution of *NFKB1* rs28362491 polymorphism was in Hardy–Weinberg equilibrium for both MI patients and control participants (all *P* > 0.05). The detected frequency of D allele and DD genotype was more common in MI patients than that in controls (D allele, 41.2% vs. 36.4%, *P* = 0.021, DD genotype, 17.5% vs. 12.0%, *P* = 0.022, Table 2). Further analysis showed that the distribution of recessive models (DD vs. ID + II, *P* = 0.007, Table 2) was significant difference between the two groups. But there was no difference in dominant models between the two groups (ID + DD vs. II, *P* = 0.159, Table 2).

**Table 2:** Frequency of *NFKB1* gene rs28362491 variation in patients with myocardial infarction (MI) and control participants.

rs28362491	Controls (n = 1085)	MI patients (n = 359)	χ^2^ value	*P* value
**Genotype**				
II, n (%)	426 (39.3)	126 (35.1)		
ID, n (%)	529 (48.7)	170 (47.4)		
DD, n (%)	130 (12.0)	63 (17.5)	7.589	0.022
**Alleles**				
I	1381 (63.6)	422 (58.8)		
D	789 (36.4)	296 (41.2)	5.447	0.021
**Recessive model**				
DD, n (%)	130 (12.0)	63 (17.5)		
II + ID, n (%)	955 (88.0)	296 (82.5)	7.220	0.007
**Dominant model**				
ID + DD, n (%)	659 (60.7)	233 (64.9)		
II, n (%)	426 (39.3)	126 (35.1)	1.982	0.159

### *NFKB1* gene DD genotype was associated with higher susceptibility to myocardial infarction

We further analyzed the potential risk factors for MI using univariate and multivariate logistic regression analysis. First, the single factor logistic regression analysis indicated that the DD genotype was the risk factor for MI (COR = 1.564, 95% CI 1.126–2.170, *P* = 0.008). Second, we utilized multivariate logistic regression analysis to examine whether the DD genotype was the real independent risk factor for MI. After eliminating the effect of traditional risk factors including sex, smoking, glucose, TC, LDL-C, HDL-C, hypertension and diabetes, DD genotype remained as an independent risk factor for MI (AOR = 1.444, 95% CI 1.01–2.051, *P* = 0.04, Table 3).

**Table 3:** Univariate and multivariate analyses of ins/del variation of *NFKB1* gene rs28362491 and other risk factors of myocardial infarction.

Risk factor	Univariate logistic regression		Multivariate logistic regression	
	COR (95% CI)	*P* value	AOR (95% CI)	*P* value
DD/ID + II	1.564 (1.126–2.170)	0.008	1.444 (1.016–2.051)	0.040
Men	1.763 (1.367–2.273)	< 0.001	1.299 (0.927–1.820)	0.129
Smoking	2.131 (1.668–2.723)	< 0.001	1.917 (1.388–2.648)	< 0.001
Glucose	1.205 (1.122–1.295)	< 0.001	1.174 (1.085–1.270)	< 0.001
TC	1.610 (1.343–1.929)	< 0.001	1.563 (1.283–1.905)	< 0.001
LDL-C	1.568 (1.266–1.941)	< 0.001	1.328 (1.056–1.670)	0.015
HDL-C	0.503 (0.283–0.893)	0.019	0.657 (0.357–1.208)	0.176
Hypertension	1.583 (1.144–2.188)	0.006	1.382 (0.947–2.017)	0.094
Diabetes	4.997 (3.115–8.016)	< 0.001	4.082 (2.399–6.947)	< 0.001

*COR* crude odds ratio, *AOR* adjusted odds ratio, *CI* confidence interval, *TC* total cholesterol, *HDL-C* high-density lipoprotein–cholesterol, *LDL-C* low density lipoprotein–cholesterol.

### Individuals with DD genotype had worse stenosis of coronary artery and inflammatory response

**Figure 2:**
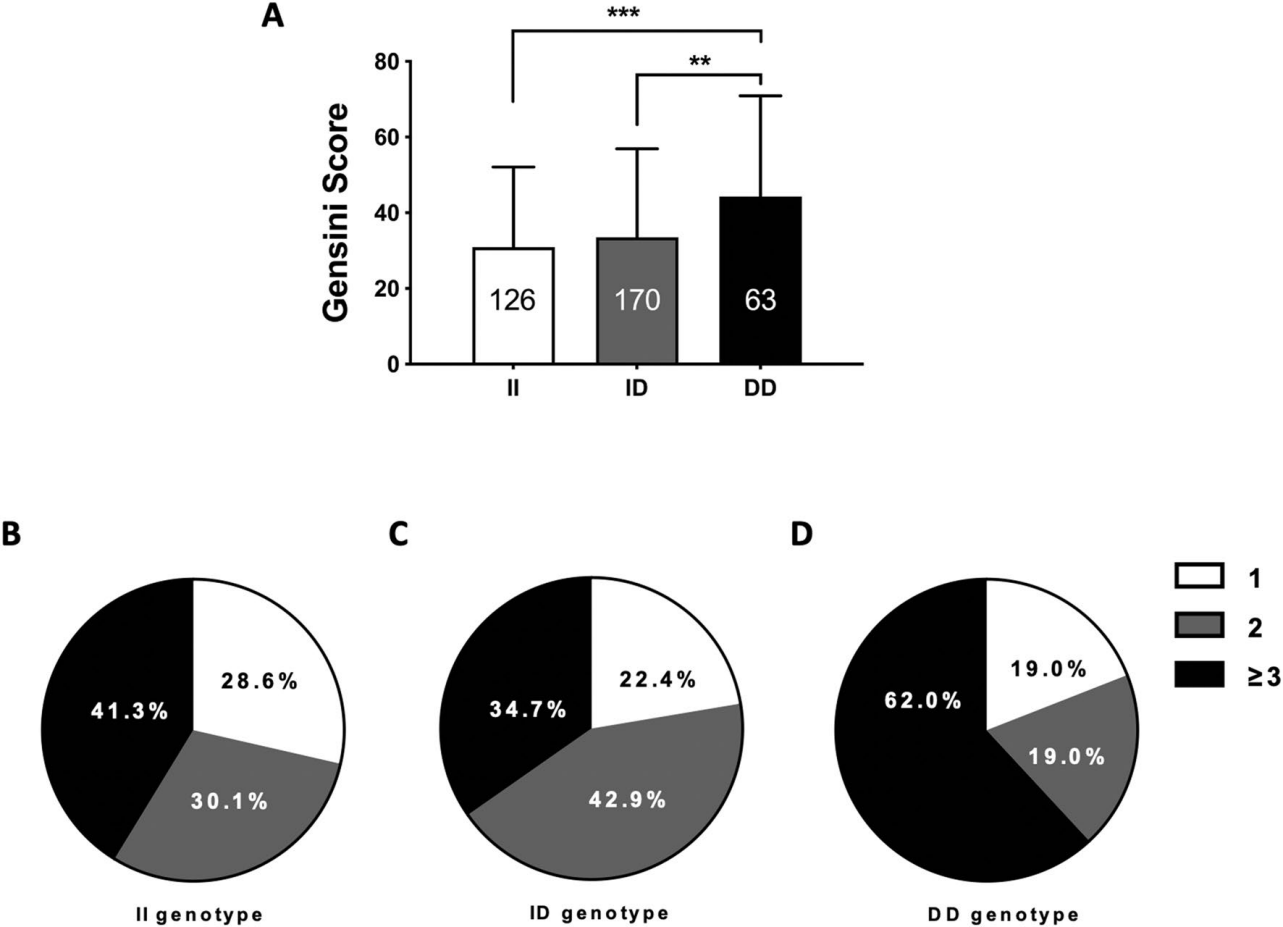
Influence of *NFKB1* gene rs28362491 variation in lesion extent of coronary artery in MI patients. (**A**) Gensini scores in MI patients with DD, II or ID genotype of *NFKB1* gene rs28362491. The numbers in bars indicate the group size. **P < 0.001, ***P < 0.0001. (**B–D**) Distribution of MI patients with the different number of diseased coronary arteries based on three different genotypes of *NFKB1* gene rs28362491. Compared with II or ID genotype carriers, Chi-square test showed that DD genotype carriers had higher percentage of triple and more triple diseased coronary artery (*P* = 0.001).

We then examined the relation between ins/del variation of *NFKB1* gene rs28362491 and the severity of coronary artery lesions among MI patients. The Gensini score was 32.2% higher in patients carrying DD genotype than that in II or ID genotype carriers (*P* < 0.001, Fig. 2A). Based on three different genotypes of *NFKB1* gene rs28362491, we analyzed the percentage of MI patients with single, double, triple and more triple diseased coronary artery. The results were showed in Fig. 2B–D. Notably, compared with II and ID genotypes, DD genotype carriers had more diseased coronary arteries (*P* = 0.001).

**Figure 3:**
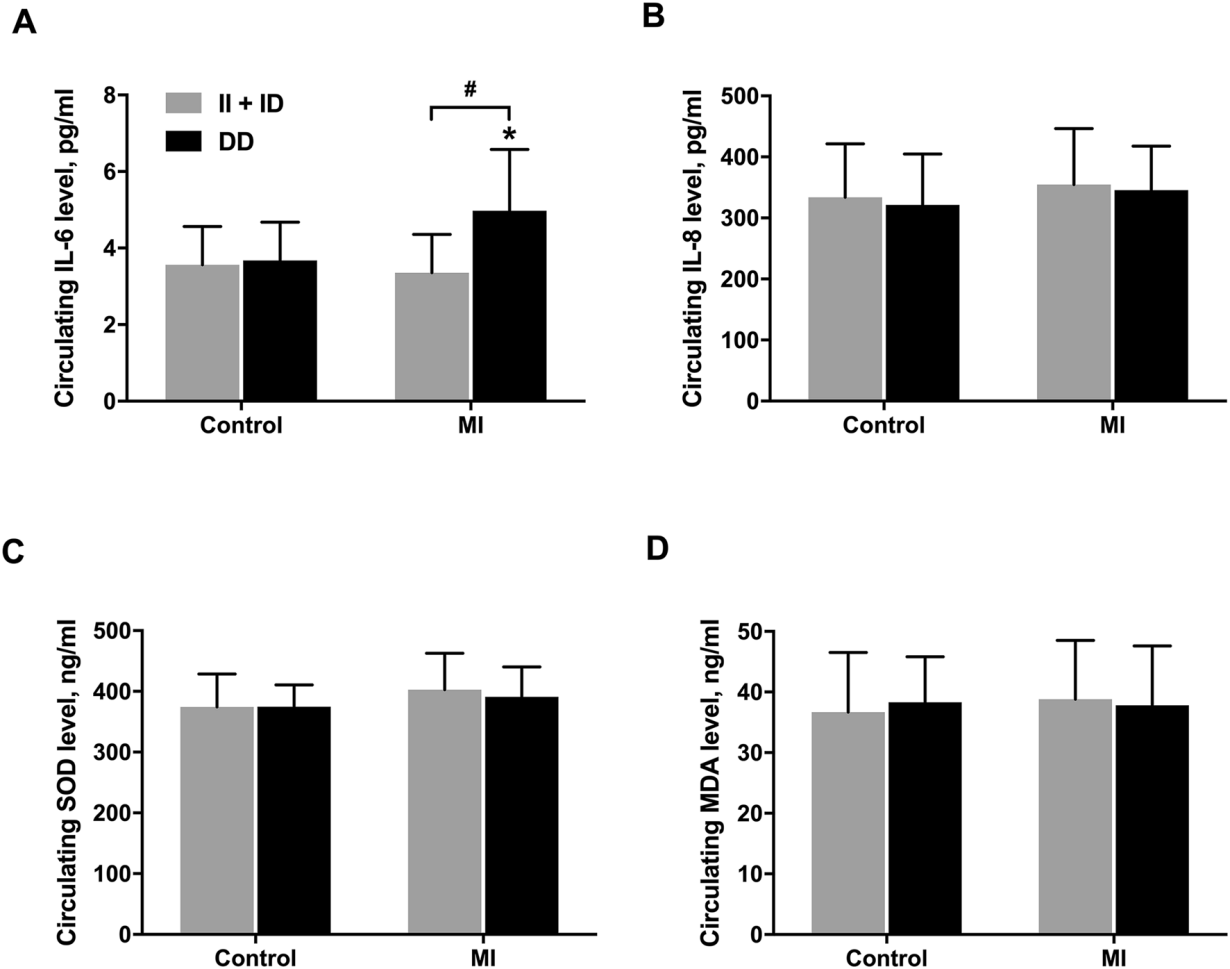
Plasma levels of IL-6, IL-8, MDA and SOD in MI patients and control participants. Plasma levels of IL-6 (A), IL-8 (**B**), MDA (**C**) and SOD (**D**) in controls participants and MI patients carrying II, ID or DD phenotype of *NFKB1* gene rs29362491. In control group, 100 participants carried II or ID genotype and 24 participants carried DD genotype. In MI group, 60 participants carried II or ID genotype and 15 participants carried DD genotype *P < 0.05 vs. all controls, ^#^*P < *0.001.

Furthermore, we measured the circulating levels of IL-6, IL-8, MDA and SOD in randomly selected participants to explore the potential influence of these variations in inflammatory cytokines and oxidative stress markers. Of note, MI patients carrying DD genotype had the highest level of IL-6 compared with II or ID genotype carriers in MI patients (*P* < 0.001) and all control participants (*P* < 0.05, Fig. 3A). However, no statistical difference was found for the levels of IL-8, MDA or SOD among different genotype carriers (Fig. 3B–D).

### Power of study

Our data indicated that the probability of exposure of *NFKB1* gene rs28362491 DD genotype in controls was 0.12 and the OR value in MI patients relative to control subjects was 1.564. The Power and Sample Size Program showed that the power of this study was 0.751, which suggested that the chance of our study based on the sample size to spot the difference between MI and control participants was 75.1%.

## Discussion

In this study, we investigated the relation between ins/del variation of *NFKB1* gene rs28362491 and the susceptibility of MI in a Chinese Han population. We found that: (1) the D allele and DD genotype were more prevalent in MI patients than in controls. (2) MI patients carrying DD genotype had higher Gensini scores and greater numbers of stenotic coronary arteries than other genotype carriers. (3) The plasma IL-6 level was higher in individuals carrying DD genotype.

The ins/del variation of *NFKB1* gene rs28362491 was firstly identified by Karban and his colleagues in 2004. They found that this variation was a susceptible factor for the ulcerative colitis^18^. Since then, several studies reported that this variation also correlated with left ventricular dysfunction^30^, dilated cardiomyopathy^31^ and heart failure^32^ in different populations. Vogel et al. reported that carriers with *NFKB1* gene rs28362491 D allele were at higher risk of CAD in Caucasians population^20^. This finding was supported by others^19,33^. Moreover, DD genotype was also be documented to be a risk factor of cardiovascular disease in rheumatoid arthritis patients in Caucasians^34^. However, Boccardi et al. reported this variation was associated with lower MI susceptibility in a small scale of case–control study conducted in southern Italy^22^. Others found the frequencies of D allele and DD genotype were comparable between MI patients and controls^23^. In addition, Jakob and his colleagues also reported that *NFKB1* gene rs28362491 variation did not associated with obesity and incident ACS^35^. Considering these contradicting reports, in our present study, we conducted a relatively large scale of case–control study to further investigate the relation between ins/del variation of *NFKB1* gene rs28362491 and MI susceptibility in Chinese Han population. Our results further documented that frequencies of D allele and DD genotype were closely associated with susceptibility of MI. This result was consistent with our previous studies. Our previous studies reported that this variation was correlated with CAD in Chinese Han and Uygur populations^15,36^. Therefore, together with these above-mentioned studies, these findings clearly indicated the *NFKB1* gene rs28362491 variation was more likely to be an independent risk factor for susceptibility of MI.

Previous studies demonstrated that ins/del variation of *NFKB1* gene rs28362491 was an essential variation with some potential function^18,37^. In the present study, we found that this variation has an association with the severity of coronary artery lesion. MI patients with DD genotype had higher Gensini scores than those with II or ID genotype. Gensini score is a well-recognized method to evaluate severity of coronary artery disease^38^. Individuals with higher Gensini score were associated with a great incident of cardiovascular events^39^. Moreover, we also observed that patients who had triple and more than triple stenotic vessels were more prevalent in patients carrying DD genotype. Our results indicate that *NFKB1* gene variation may have a potential impact on the coronary artery lesion.

NF-κB is an inducible cytoplasmatic transcriptional factor which transfers into the nucleus upon activation and then binds to the promoter regions of many genes including proinflammtory genes such as IL-1, IL-6, IL-8 and TNF-α and increases their expression^8,9^. IL-6, an important proinflammatory cytokine, is a central mediator of inflammatory process ^40^. Increased circulating level of IL-6 has been reported in many inflammatory diseases, especially, in atherosclerosis, CAD, acute MI and metabolic syndromes^41^. IL-8 is also a proinflammatory cytokine that is expressed in macrophage-rich areas of atherosclerotic lesions^42^. Elevated levels of IL-8 were associated with an increased risk of CAD^43,44^. Circulating levels of SOD and MDA can be measured to assess the degree of oxidative stress^11,12^. In this study, we measured plasma levels of IL-6, IL-8, MDA and SOD to investigate whether *NFKB1* gene rs28362491 variation would affect their expression. Albeit we did not detect difference in the levels of IL-8, MDA and SOD among three phenotypes carriers, we observed that plasma IL-6 levels were significantly higher in individuals with DD genotype than those with other genotypes in MI patients and all control participants. The highest level of IL-6 was detected in MI patients with DD phenotype. This result was similar to our previous study which showed the plasma IL-6 level was significantly higher in the stable angina pectoris patients with DD genotype in Chinese Uygur population^15^. Other clinical studies documented that the high IL-6 level is an independent risk factor for CAD and chronic heart failure^45,46^. In addition, this variation affected IL-6 level in patients with Hashimoto thyroiditis in a Turkish population^47^. Taken together, these results suggested that *NFKB1* gene rs28362491 variation had a potential influence on the expression of IL-6.

There were several limitations in our study. First, we focused on the association between *NFKB1* gene rs28362491 variation and susceptibility of MI. Whether this variation affects the prognosis of both MI patients and controls is still not clear. A relatively large and long-term follow-up study is necessary to investigate its influence in the future. Second, although we have found that *NFKB1* gene rs28362491 variation affected circulating IL-6 level, which might be the potential mechanism of this variation on susceptibility of MI, the exactly mechanism should be verified in vivo or in vitro. Third*,* inflammatory and oxidative stress biomarkers were measured only in a small proportion of participants, which would mask their significance. Together with other’s positive findings, these results indicate a potential clinic significance. Further gene sequencing for individuals with traditional risk factors could help to identify whether carrying *NFKB1* DD genotype, which may help clinician to take more active interventions to reduce risk of MI. This is also the real purpose of precision medicine.

## Conclusion

Our study documented *NFKB1* gene rs28362491 DD genotype was associated with higher risk of MI and more severe of coronary artery lesion. This variation also has a functional influence in circulating IL-6 level.

## Supplementary information

Supplementary Information

## Abbreviations

MI: Myocardial infarction
ACE: Angiotensin-converting enzyme
SLC6A4: Serotonin transporter
eNOS: Endothelial nitric oxide synthase
NF-κB: Nuclear factor κB
IL-6: Interleukin-6
IL-8: Interleukin-8
MDA: Malonaldehyde
SOD: Superoxide dismutase
II genotype: Wild-type homozygous insertion
DD genotype: Variant homozygous deletion
ID genotype: Heterozygous
CAD: Coronary artery disease
LAD: Left anterior descending
LCX: Circumflex artery
RCA: Right coronary artery
TC: Total cholesterol
TG: Triglycerides
LDL-C: Low density lipoprotein-cholesterol
HDL-C: High density lipoprotein-cholesterol
BMI: Body mass index
SBP: Systolic blood pressure
DBP: Diastolic blood pressure
PCR-RFLP: Polymerase chain reaction–restriction fragment length polymorphism
ELISA: Enzyme linked immunosorbent assay
SD: Standard deviation
COR: Crude odds ratios
AOR: Adjusted odds ratios
