# Supplementary material for: NFKB1 gene rs28362491 ins/del variation is associated with higher susceptibility to myocardial infarction in a Chinese Han population
Source: Sci Rep. 2020 Nov 11;10:19518. doi: 10.1038/s41598-020-72877-9 (PMC7658993; doi:10.1038/s41598-020-72877-9)
Supplement: Supplementary file 1 — Supplementary Information [file 41598_2020_72877_MOESM1_ESM.docx]

**Cover page**

***NFKB1* gene rs28362491 ins/del variation is associated with higher** **susceptibility to myocardial infarction in a Chinese Han population**

Jun-Yi Luo^1,^*, Yan-Hong Li^2,^*, Bin-Bin Fang^3^, Ting Tian^1^, Fen Liu^3^, , Xiao-Mei Li^1^, Xiao-Ming Gao^3,4^ #, Yi-Ning Yang^1^#

^1^ *State Key Laboratory of Pathogenesis, Prevention and Treatment of High Incidence Diseases in Central Asian. Department of Cardiology, First Affiliated Hospital of Xinjiang Medical University, Urumqi, China*

*^2^ Department of Medical Science Examination Center,* *First Affiliated Hospital of Xinjiang Medical University, Urumqi, Xinjiang, China*

*^3^ State Key Laboratory of Pathogenesis, Prevention and Treatment of High Incidence Diseases in Central Asian, First Affiliated Hospital of Xinjiang Medical University, Urumqi**, China*

*^4^ Xinjiang Key Laboratory of Medical Animal Model Research, Urumqi, China*

***Short title: NFKB1 gene and MI susceptibility***

* Jun-Yi Luo and Yan-Hong Li contributed equally to this manuscript.

Jun-Yi Luo: luojunyi3130@126.com

Yan-Hong Li: yanhongli1225@163.com

Bin-Bin Fang: xj_binbin5515@sina.com

Ting Tian: cherrytian830@hotmail.com

Fen Liu: 604760840@qq.com

Xiao-Mei Li: lixm505@163.com

Xiao-Ming Gao: xiaominggao2017@163.com

Yi-Ning Yang: yangyn5126@163.com

**# Corresponding author**

Prof. Yi-Ning Yang

Department of Cardiology, First Affiliated Hospital Xinjiang Medical University, 137 Liyushan South Road, Urumqi, 830054, Xinjiang, China.

Tel: +86-991-4362611 Fax: +86-991-4365381 E-mail: yangyn5126@163.com

Prof. Xiao-Ming Gao

State Key Laboratory of Pathogenesis, Prevention and Treatment of High Incidence Diseases in Central Asian. 137 Liyushan South Road, Urumqi, 830054, Xinjiang, China.

Tel: +86-991-4362844, Fax: +86-991-4362844, Email: xiaomingao2017@163.com.

**Supplementary figure legends**

**Supplementary figure 1. The** **original image of polymerase chain reaction-restriction fragment length polymorphism analysis for genotyping *NFKB1* rs28362491 variation.**

**
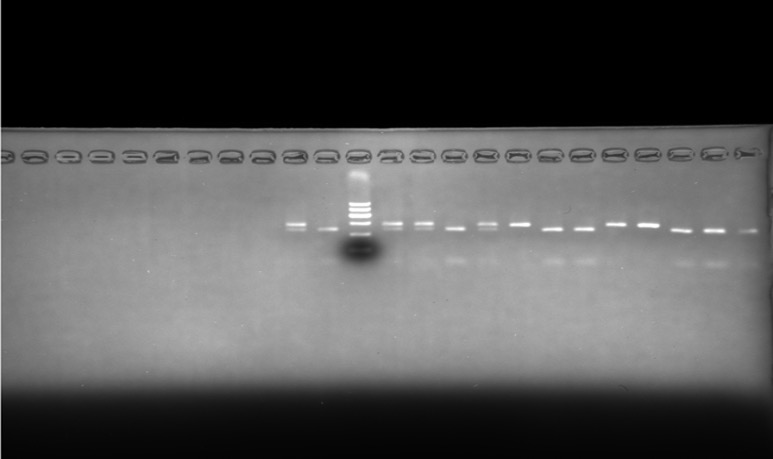
**
